# Obstructive sleep apnea and nocturnal enuresis in the pediatric population: a systematic review and meta-analysis

**DOI:** 10.1007/s11325-026-03609-y

**Published:** 2026-02-20

**Authors:** Adam Y. Diab, Tala AlNatsheh, Sham Tkwa, Saja Al-Juboori, Noor Jaber, Ahmad A. Toubasi

**Affiliations:** 1 School of Medicine, The University of Jordan, Amman, 11942 Jordan; 2 Department of Neurology, Vanderbilt University Medical Center (VUMC), Nashville, TN USA; 3 Rachid lab, Department of Allergy & Immunology, Harvard School, Boston Children’s Hospital, Boston, MA United States

**Keywords:** Bedwetting, Enuresis and OSA, Nocturnal enuresis, Obstructive sleep apnea, Polysomnography, Sleep-disordered breathing, Systematic review

## Abstract

**Background:**

The relationship between nocturnal enuresis (NE) and obstructive sleep apnea (OSA) among the pediatrics population is considered complex. We aimed to assess the prevalence of NE in patients with OSA and the potential relationship between the two.

**Methods:**

We searched PubMed, Scopus, Web of Science, and Cochrane up to October 2024 using terms related to OSA and NE. Eligible studies were observational, published in English, involved participants under 18, and examined the OSA–NE association.

**Results:**

The total number of participants included was 11,612 from 15 articles. Twelve studies reported the prevalence of enuresis among children with OSA, with a pooled prevalence of 40% (95% CI: 30%–51%). In studies using polysomnography, prevalence was 43% (95% CI: 32%–53%). Eight studies assessed the association between OSA and enuresis, showing a significant relationship (OR = 2.30; 95% CI: 1.37–3.84), which remained consistent in the polysomnography subgroup (OR = 2.62; 95% CI: 1.53–4.48). Enuretic patients had higher AHI (WMD = 3.37; 95% CI: 0.61–6.14) and BMI (WMD = 0.90; 95% CI: 0.74–1.06), but no significant differences were found in total sleep time or REM sleep.

**Conclusion:**

This systematic review reveals a significant association between OSA and enuresis in children, with a high prevalence of enuresis among those with OSA. The findings highlight the importance of screening for sleep-disordered breathing in children with persistent enuresis. Further research is needed to establish causality and evaluate the impact of OSA treatment on enuresis resolution.

## Introduction

Nocturnal enuresis (NE), or bedwetting, is the involuntary loss of urine that happens during sleep in children ages five and older. It is classified as primary (never dry for six months) or secondary (recurrence after dryness). NE affects 15% of 5-year-olds, decreasing with age, and is more common in boys [1].

Risk factors include delayed bladder control, high nighttime urine production, deep sleep, constipation, and family history [2]. While not a serious medical issue, it can impact self-esteem and social life [3].

Obstructive sleep apnea (OSA) is a sleep-related breathing disorder (SRBD) characterized by recurrent episodes of partial or complete upper airway obstruction during sleep, leading to disrupted sleep and oxygen de-saturation. It is more prevalent in children with enlarged tonsils, obesity, or craniofacial abnormalities [4]. OSA affects approximately 1–5% of children, with peak prevalence between ages 2 and 8 years [5].

Risk factors include adenotonsillar hypertrophy, obesity, neuromuscular disorders, and family history [6]. If left untreated, OSA can result in behavioral problems, cognitive impairment, cardiovascular complications, and metabolic disturbances [4]. Early diagnosis and intervention, including adenotonsillectomy, weight management, and continuous positive airway pressure (CPAP) therapy, are essential to prevent long-term consequences.

The correlation between NE and OSA in children is both clinical and theoretical, with evidence documented in the literature [7, 8] and recognized by experts for a long time. This connection is attributed to several factors, including physiologic plausibility due to shared mechanisms. For instance, the rise in atrial natriuretic peptide (ANP) resulting from exaggerated and repeated intrathoracic pressure swings plays a significant role [9, 10]. Additionally, other hypothesized mechanisms link the two disorders, such as arousal dysfunction, where OSA-induced fragmented sleep impairs the arousal response necessary to wake up and void [11, 12], and autonomic dysregulation, which affects both bladder function and arousal, contributing to the overlap between the conditions [13]. The strongest evidence connecting the two disorders is the resolution of enuresis symptoms following effective treatment of OSA, whether through adenotonsillectomy, weight loss, or other therapeutic approaches [7, 14, 15].

Prior intervention-based meta-analyses Such as Wang et al. (2025) [16] demonstrated the efficacy of some interventions that relieves OSA or improve it also improve or completely resolve enuresis indicating and indirect relationship between OSA and enuresis.

While some studies suggest the potential association, the exact mechanisms linking the two conditions are not fully understood with much of the existing literature being limited by small sample sizes or retrospective designs, highlighting the need for larger, more rigorous research to better clarify the nature and extent of this relationship [11, 12].

In this systematic review, we aim to explore the potential relationship between NE and OSA in children. We hypothesize that there is a significant association between these two conditions.

## Materials and methods

### Search strategy

Up to October 2024, we systematically searched the following databases: PubMed, Scopus, and Web of Science Cochrane. Combinations of obstructive sleep apnea-related medical subject headings (MeSH) terms (Obstructive sleep apnea OR OSA OR OSAHS OR Sleep apnea–hypopnea syndrome OR Obstructive sleep apnea syndrome OR Upper airway resistance sleep apnea syndrome) and nocturnal enuresis-related MeSH terms (Nocturnal Enuresis OR Bedwetting OR Nighttime Urinary Incontinence) were employed. The search results were uploaded to Rayyan, a web-based application for systematic reviews, and duplicates were removed.

### Inclusion criteria and exclusion criteria

The following inclusion criteria were applied to the literature search results: (1) the article is a cross-sectional, case–control, or cohort published in English language journals; (2) the age group studied was those younger than 18 and (3) the study evaluated the association between OSA and NE. Reviews and case reports were excluded, in addition to articles that investigated each disorder alone and without analyzing the correlation between the two. Articles on the impact of OSA treatment methods on NE were also excluded. We also excluded articles that studied the effect of NE on the symptoms of OSA. The study selection was done by A.D, T.A, SA, N.J and S.T and any disagreement was solved by the senior investigator (A.A.T).

### Data extraction

Data extraction was performed on a standard data extraction form provided. The variables extracted from the included articles were: authors information, study design, country where the study was conducted, sample size, age of the included participants, number of males and females, the method used to evaluate OSA, body mass index (BMI) of patients with and without nocturnal enuresis, apnea–hypopnea index (AHI) of patients with and without nocturnal enuresis, total sleep time in minutes of patients with and without nocturnal enuresis, rapid eye movement (REM) sleep time of patients with and without nocturnal enuresis. The full-text data extraction was done by A.D, A.T, T.A, S.A, and S.T. Disagreements were resolved by A.A.T until a mutual consensus was achieved.

### Assessment of study quality

The quality of studies included in this review were assessed by the Newcastle–Ottawa Scale (NOS) as a tool designed to assess the quality of non-randomized studies included in a systematic review and/or meta-analyses, as each study was evaluated based on nine items which were classified into three groups: selection of the study groups, comparability and the outcome. A study was defined as high quality if it got a score of higher than 5.

### Statistical analysis

The statistical analysis was done using Meta-XL Epigear v5.3. Odds ratio (OR) and its related 95% confidence intervals (95%CI) were used as the effect measure for binary associations. The weighted mean difference (WMD) and its 95%CIs were used as the effect measure for continuous variables. The random effect model was used when the heterogeneity was ≥ 50% while the fixed effect model was used when the heterogeneity was < 50%. Subgroup analysis among studies that used polysomnography to diagnose OSA and among good quality studies (NOS > 5) were conducted.

## Results

### Search results

**Fig. 1:**
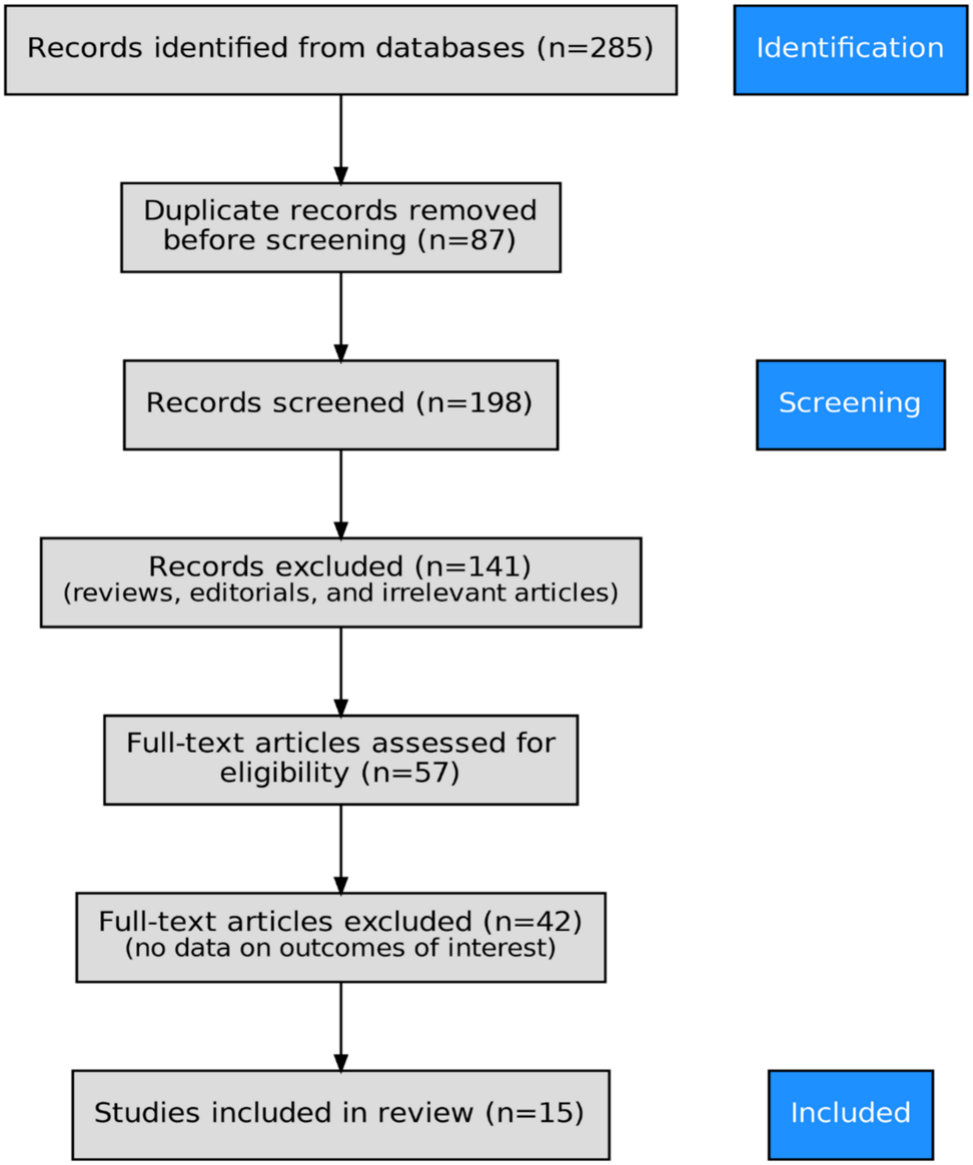
PRISMA 2020 Flow Diagram Showing the Study Selection Process

The search yielded 285 articles. Of them, 87 were duplicates. The remaining (n = 198) articles were screened based on their title and abstract and 141 articles were excluded because they were reviews, editorials, and irrelevant. The lasting 57 articles were reviewed in their full-text form and 42 were excluded because they did not have data about the outcomes of interest. Finally, 15 articles were included in our systematic review and meta-analysis. Figure 1 demonstrates the study selection process.

### Characteristics of the included articles

The total number of included participants was 11,612 from the included 15 articles. Five of these articles were cross-sectional while the rest were cohort or case–control studies. The majority of the studies were conducted in Europe (n = 6) and America (n = 5) with only two studies conducted in Asia and Africa. The mean age of the included participants was 7.98 ± 2.80. In addition, 55.3% of the participants were males while the rest were females. The majority of the included studies were of good quality (NOS > 5) (73.3%). Table 1 demonstrate the characteristics of the included articles.

**Table 1:** The characteristics of the included studies

Study	Design	Country	Sample Size	NOS
(Sakellaropoulou et al., 2012)	Retrospective cohort	Greece	52	3
(Barone et al., 2009)	Case–control	United States of America	174	5
(Martenstyn et al. 2021)	Case–control	Australia	378	5
(Fernandes et al., 2018)	Cross-sectional	Brazil	153	5
(Capdevila et al. 2008)	Cross-sectional	United States of America	525	6
(Waleed et al., 2011)	Cross-sectional	Egypt	160	9
(Alexopoulos et al., 2006)	Retrospective cohort	Greece	8616	9
(Brooks & Topol, 2003)	Cross-sectional	United States of America	221	9
(Tsai et al., 2017)	Case–control	Taiwan	66	7
(Lehmann et al., 2012)	Prospective cohort	United States of America	41	8
(Kol et al., 2024)	Prospective cohort	Turkey	525	6
(Ring et al., 2017)	Case–control	Sweden	73	8
(Alexopoulos et al., 2014)	Cross-sectional	Greece	298	9
(El-Mitwalli et al. 2014)	Case–control	Egypt	52	9
(Andreu-Codina et al., 2024)	Prospective cohort	Spain	174	9

### Prevalence of enuresis among patients with OSA

**Fig. 2:**
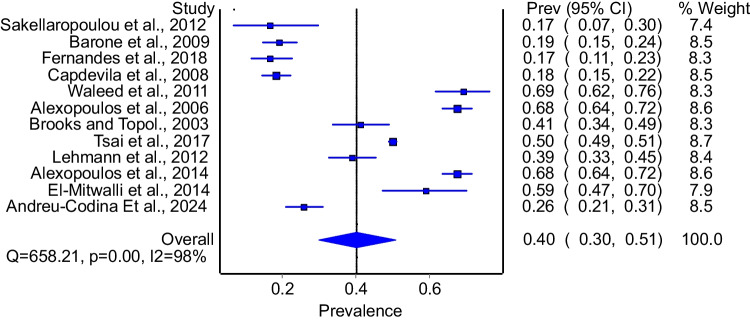
Prevalence of enuresis among patients with OSA

**Fig. 3:**
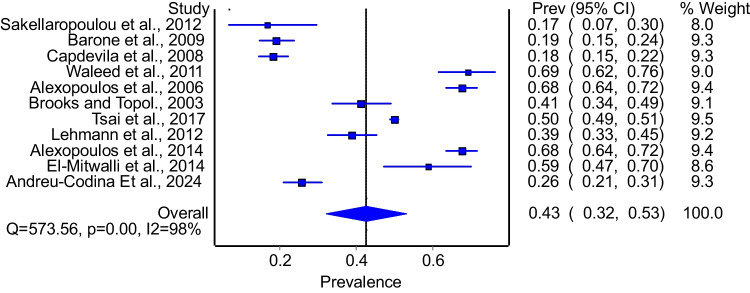
Prevalence of enuresis among patients with OSA among studies that diagnosed OSA using polysomnography

Twelve studies evaluated the prevalence of enuresis among patients with OSA. The model demonstrated that 40% of patients with OSA had enuresis (Fig. 2: 95%CI: 30%−51%). The model had significant heterogeneity (I^2^ = 98%, p = 0.00). Subgroup analysis that included studies that used polysomnography to diagnose OSA included 11 studies. The model that pooled these studies demonstrated a prevalence of 43% (Fig. 3: 95%CI: 32%−53%) with significant heterogeneity (I^2^ = 98%, p = 0.00). Subgroup analysis of good quality studies included 9 studies and the model demonstrated a prevalence of 48% (95%CI: 38%−59%) with significant heterogeneity (I^2^ = 98%, p = 0.00).

### The association between enuresis and OSA

**Fig. 4:**
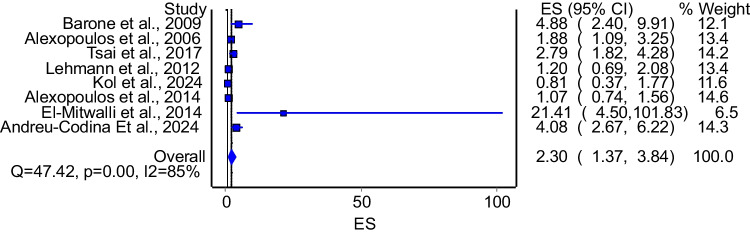
The association between OSA and enuresis

**Fig. 5:**
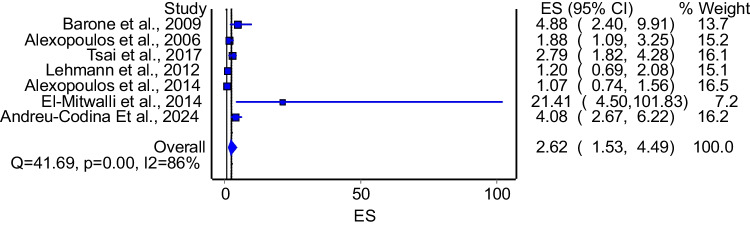
The association between OSA and enuresis among studies that diagnosed OSA using polysomnography

The model that evaluated the association between enuresis and OSA included 8 studies. The model demonstrated significant association between OSA and enuresis (Fig. 4: OR = 2.30; 95%CI: 1.37–3.84). The model had significant heterogeneity (I^2^ = 85%, p = 0.00). A subgroup analysis that included studies that used polysomnography to diagnose OSA included 7 studies. The model that pooled these studies also demonstrated significant association between OSA and enuresis (Fig. 5: OR = 2.62; 95%CI: 1.53–4.48). The model had significant heterogeneity (I^2^ = 86%, p = 0.00). Subgroup analysis of good quality studies included 7 studies and the model showed a significant association between OSA and enuresis (OR = 2.06; 95%CI: 1.20–3.54). The model had significant heterogeneity (I^2^ = 85%, p = 0.00).

### OSA related characteristics and its association with enuresis

**Fig. 6:**
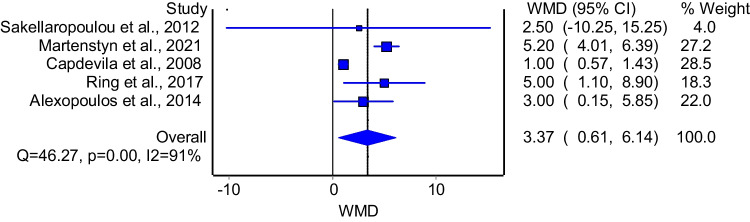
The association between AHI and enuresis

The model that investigated the association between AHI and enuresis demonstrated that patients with enuresis had significantly higher AHI compared to their counterparts (Fig. 6: WMD = 3.37; 95%CI: 0.61–6.14). The model had significant heterogeneity (I^2^ = 91%, p = 0.00). Subgroup analysis of good quality studies included three studies and demonstrated consistent results with the primary analysis (WMD = 2.40; 95%CI: 0.11–4.69); the model had insignificant heterogeneity (I^2^ = 65%, p = 0.06).

**Fig. 7:**
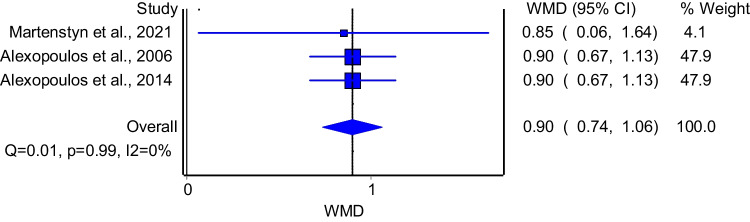
The association between BMI and enuresis

Three studies evaluated the association between BMI and enuresis. The model that pooled these studies demonstrated that patients with enuresis had significantly higher BMI compared to their counterparts (Fig. 7: WMD = 0.90; 95%CI: 0.74–1.06). The model had insignificant heterogeneity (I^2^ = 0%, p = 0.99). Subgroup analysis of good quality studies included two papers and showed consistent results with the primary analysis (WMD = 0.90; 95%CI: 0.74–1.06); the model had insignificant heterogeneity (I^2^ = 0%, p = 1.00).

**Fig. 8:**
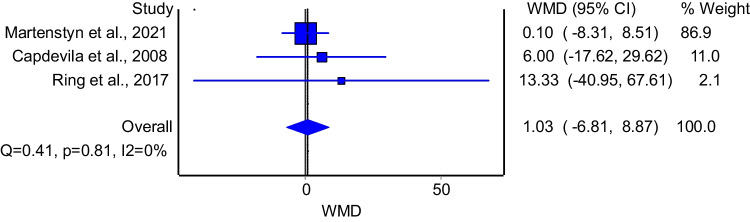
The association between total sleep time and enuresis

Three studies evaluated the difference in total sleep time between patients with and without enuresis. The model that pooled these studies demonstrated no significant difference between the two groups (Fig. 8: WMD = 1.03; 95%CI: −6.81–8.87). The model had insignificant heterogeneity (I^2^ = 0%, p = 0.81). Subgroup analysis of good quality studies included two papers and showed similar results to the primary analysis (WMD = 7.17; 95%CI: −14.49 −28.82); the model had insignificant heterogeneity (I^2^ = 0%, p = 0.81).

**Fig. 9:**
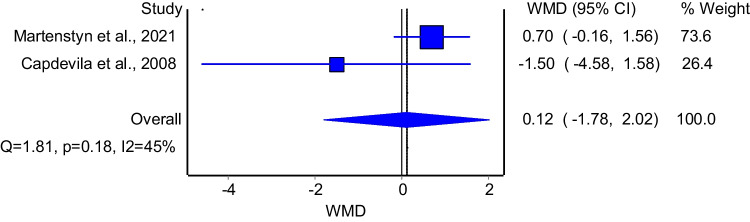
The association between REM sleep time and enuresis

The model that evaluated the association between REM sleep time and enuresis included 2 studies. The model found no significant difference in REM sleep time between patients with and without enuresis (Fig. 9: WMD = 0.12; 95%CI: −1.78–2.02); the model had insignificant heterogeneity (I^2^ = 45%, p = 0.18). Subgroup analysis among good quality studies was not possible due to the low number of the included studies.

## Discussion

This systematic review and meta-analysis included 15 studies with 11,612 participants, primarily from Europe and America. The prevalence of enuresis among OSA patients was estimated at 40%, which slightly increased 43% in studies using polysomnography. A significant association was found between OSA and enuresis, which remained significant when only studies using polysomnography were included. Patients with enuresis had significantly higher AHI and BMI compared to those without enuresis. However, no significant differences were observed in total sleep time or REM sleep time between the two groups. Subgroup analysis among good quality studies (NOS > 5) confirmed these findings.

The overall prevalence of enuresis we found among OSA patients was estimated at 40% with high heterogeneity between the included studies. This high heterogeneity might be due to several reasons among the included studies such as different study designs, characteristics of the included populations, and definitions of the exposures and outcomes. El-Mitwalli et al. (2014) [17] reported the highest prevalence of enuresis among patients with enuresis, most likely as a result of several important factors. The gold standard for diagnosing OSA, polysomnography, was employed in the study to ensure precise diagnosis of sleep-disordered breathing (SDB). Children with refractory monosymptomatic NE were also included in the study cohort. The link between enuresis and sleep apnoea may be strengthened in these kids because they are more likely to have underlying medical issues, such as severe OSA. Additionally, because the study included patients from a neurology outpatient clinic—a facility that usually handles more difficult cases—selection bias might have played a role in the high prevalence of enuresis. **Capdevila et al**. (2008) [18] and **Fernandes et al**. (2018) [19] were among the studies reporting the lowest prevalence of enuresis among patients with OSA, mostly as a result of variations in the populations sampled and the methods used to evaluate OSA. Instead of using a clinical group with severe OSA, **Capdevila et al**. (2008) [18] used a large community-based survey that included children with lesser forms of SDB. Given that studies with higher prevalence usually include children with moderate-to-severe OSA, this wide sample strategy probably contributed to reducing the observed prevalence of enuresis. Additionally, only frequent bedwetting (≥ 3 nights per week) was taken into consideration in this study, which may have excluded children with occasional enuresis and further reduced the reported prevalence. This is also true of **Fernandes et al**. (2018) [19], which employed a questionnaire-based technique called the sleep disturbance scale for children instead of polysomnography, the gold standard for diagnosing OSA. Because subjective screening was used instead of objective sleep investigations, children with actual OSA might have been misclassified, weakening the link between OSA and enuresis. The high prevalence of enuresis among the included studies highlight the significance of the disorder as it has several life-changing consequences. Psychosocially, it can lead to low self-esteem, embarrassment, and social withdrawal, particularly in older children [20]. It also contributes to sleep disruption, as frequent nighttime awakenings due to bedwetting can further fragment sleep already disturbed by OSA, worsening daytime sleepiness [21]. Additionally, cognitive and behavioral effects have been observed, with studies suggesting that children experiencing both enuresis and OSA may be at higher risk for attention deficits, hyperactivity, and academic struggles compared to those with OSA alone [22]. Finally, enuresis imposes a significant burden on families, increasing parental stress and health [23].

Our systemic -analysis also demonstrated that individuals with OSA had significantly higher odds of experiencing NE, which is consistent with Alexopoulos et al. [24], who found a strong correlation between moderate-to-severe OSA and NE. Moreover, our subgroup analysis of polysomnography-based studies (OR = 2.62; 95% CI: 1.53–4.48) further supports this association, aligning with the work of Park et al. [25] and Wang et al. This also aligns with findings from studies by Park et al. [25], Tsai et al. [26], and Lumeng et al. [5], all of whom reported increased bed-wetting frequency in children with severe OSA. These results can be explained by the study conducted by Ritting et al., [27] which suggested that children with OSA experience periods of increased negative intrathoracic pressure due to enhanced inspiratory effort, leading to cardiac distention. This distention triggers the release of ANP, which may contribute to enuresis. An alternative theory proposes that children with OSA have impaired secretion of antidiuretic hormone (ADH), resulting in increased nocturnal urine volume. This disruption in normal hormone secretion, along with alterations to the stable circadian rhythm caused by the hypoxic environment of OSA, may prevent children from waking up during the bladder filling process. Consequently, this inability to wake, despite bladder contractions signaling the need for micturition, leads to NE [28].

Regarding OSA severity, our analysis revealed that children with NE had significantly higher AHI values (WMD = 2.87; 95% CI: 0.39–5.36), consistent with previous studies by Alexopoulos et al. [15] and Lumeng et al. [5]. These results suggest that the severity of OSA may play an important role in the pathophysiology of NE. However, the presence of inconsistencies across studies indicates that other factors may contribute to this relationship.

In terms of obesity, we found that children with NE had significantly higher BMI values compared to those without NE. This finding is consistent with research by Barone et al. [29] and Su et al. [30], who highlighted that obesity exacerbates both the risk of OSA and NE. This observation underscores the potential role of additional factors, such as snoring or hormonal imbalances, in contributing to the association between OSA and NE.

Lastly, our study did not find significant differences in total sleep time, REM sleep time, or sleep architecture between children with and without NE, which contrasts with findings by Zhu et al. [31], who reported that children with primary nocturnal enuresis (PNE) spent more time in light sleep and had a higher arousal index compared to controls. They also noted altered thalamocortical functional connectivity in children with NE. However, it is important to highlight that our analysis included a low number of studies and patients which might reduce our power to draw firm conclusions about sleep characteristics and NE. This discrepancy suggests that further research is needed to better understand the role of sleep stages in the development of NE in children with OSA. This is important to solve the puzzle of this complex relationship to lead to better management of the condition.

In addition to observational evidence, the association between OSA and nocturnal enuresis is further supported by interventional studies examining the effect of OSA treatment on enuresis outcomes. A recent systematic review and meta-analysis of intervention studies demonstrated that a substantial proportion of children with OSA experience partial or complete resolution of enuresis following treatment, most commonly adenotonsillectomy, with reported remission rates exceeding 50% in several cohorts [16]. Similar findings have been consistently reported across prospective and retrospective interventional studies, which show significant reductions in enuresis frequency after surgical or non-surgical management of sleep-disordered breathing, including continuous positive airway pressure (CPAP) [32]. These results provide indirect but compelling evidence for a causal relationship, suggesting that relief of upper airway obstruction and normalization of sleep-related physiologic disturbances, such as intrathoracic pressure swings, nocturnal polyuria, and impaired arousal mechanisms, may lead to improvement or resolution of enuresis. Although the present review focused exclusively on observational studies and therefore did not directly assess treatment effects, the convergence of observational associations with interventional improvements strengthens the biological and clinical plausibility of the OSA–NE relationship and highlights the importance of integrating therapeutic evidence into future research frameworks.

This systematic review has several limitations that should be acknowledged, many of which stem from the characteristics of the included studies themselves. This review focused exclusively on pediatric populations, which limited our generalizability to adults and adolescents. While this approach was both justified and intentional to increase the homogeneity in our population, it excludes insights from older populations, where the relationship between OSA and enuresis may differ due to factors such as pubertal hormonal changes or different anatomical risk factors for OSA. Additionally, many of our studies lacked demographic data such as socioeconomic status and nutritional status which are important risk factors for both OSA and enuresis in the pediatric population due to associated factors such as stress and suboptimal sleeping environment. Moreover, children with complex medical histories (e.g., neurodevelopmental disorders, prematurity, congenital urological anomalies) were either excluded from some studies or not explicitly accounted for in the analysis. There was also considerable variability in the methods used to diagnose OSA across studies. For example, Sakellaropoulou et al., 2012 and Barone et al., 2009 utilized overnight polysomnography — the diagnostic gold standard. In contrast, Bascom et al., 2011 used the OSA-18 quality of life questionnaire. Subjective tools are prone to recall bias and parental over- or under-reporting, reducing the diagnostic consistency across the included studies and increasing the risk of misclassification bias. Additionally, none of the included studies applied a standardized OSA severity threshold across the board. For example, some defined OSA based on a specific AHI cutoff (e.g., > 1 event/hour in Sakellaropoulou et al., 2012), while others used less precise definitions such as “clinical diagnosis of OSA” derived from history and symptoms. This lack of a uniform severity definition further complicates any attempt to explore dose–response relationships between OSA severity and enuresis frequency — a potentially valuable insight that this review could not capture. The definition and diagnostic criteria for enuresis also lacked consistency across studies. Sakellaropoulou et al., 2012 defined enuresis as at least one episode per week, while Barone et al., 2009 specifically included only monosymptomatic nocturnal enuresis. This lack of standardized definitions introduces measurement bias and limits the ability to compare enuresis prevalence across studies. Key variables, such as total sleep time, REM sleep duration, and frequency of enuresis episodes, were incompletely reported across several studies. This data incompleteness prevented further exploration of this complex relationship, limiting deeper insights on the topic analysis. Finally, some of our models had high heterogeneity which limits our findings. Despite conducting sensitivity analysis based on the papers that have done polysomnography and good quality papers, the heterogeneity did not come down. We also tried conducting jackknife analysis by excluding studies from the models sequentially, however the heterogeneity did not drop indicating equal distribution of the heterogeneity across the included studies. In such situations, heterogeneity is attributed to factors related to the demographics of the included patients such as heterogenous age, sex or race distributions. Due to the low number of papers, regression analysis was not possible unfortunately. Future studies with larger sample sizes might be able to address sources of heterogeneity through sensitivity analysis or meta-regression.

In conclusion, this systematic review and meta-analysis highlights a significant association between OSA and enuresis in children. Our findings reveal that enuresis is highly prevalent among pedatrics with OSA. The strong association between OSA and enuresis underscores the need for routine screening for SDB in children with persistent or treatment-resistant enuresis. Given these findings, healthcare providers should incorporate sleep history into enuresis evaluations, consider polysomnography in high-risk cases, and explore OSA-targeted treatments like adenotonsillectomy, CPAP, and weight management. Further research is needed to establish causality and evaluate the impact of OSA treatment on enuresis resolution. Standardizing diagnostic criteria will improve study comparability and clinical decision-making.

## Data Availability

The data associated with this manuscript is available from the corresponding author upon reasonable request.
